# Spatio-functional organization in virocells of small uncultivated archaea from the deep biosphere

**DOI:** 10.1038/s41396-023-01474-1

**Published:** 2023-07-19

**Authors:** Indra Banas, Sarah P. Esser, Victoria Turzynski, André Soares, Polina Novikova, Patrick May, Cristina Moraru, Mike Hasenberg, Janina Rahlff, Paul Wilmes, Andreas Klingl, Alexander J. Probst

**Affiliations:** 1 Environmental Metagenomics, Research Center One Health Ruhr of the University Alliance Ruhr, Faculty of Chemistry, University of Duisburg-Essen, Essen, Germany; 2 Group for Aquatic Microbial Ecology, Environmental Microbiology and Biotechnology, Faculty of Chemistry, University Duisburg-Essen, Essen, Germany; 3 Luxembourg Centre for Systems Biomedicine, University of Luxembourg, Esch-sur-Alzette, Luxembourg; 4 Institute for Chemistry and Biology of the Marine Environment (ICBM), Carl-von-Ossietzky-University Oldenburg, Oldenburg, Germany; 5 Imaging Center Essen, EMU, Essen, Germany; 6 Department of Life Sciences and Medicine, Faculty of Science, Technology and Medicine, University of Luxembourg, Belvaux, Luxembourg; 7 Plant Development & Electron Microscopy, Biocenter LMU Munich, Planegg-Martinsried, Planegg, Germany; 8 Centre of Water and Environmental Research (ZWU), University of Duisburg-Essen, Essen, Germany; 9 Center of Medical Biotechnology (ZMB), University of Duisburg-Essen, Essen, Germany; 10 Present Address: Centre for Ecology and Evolution in Microbial Model Systems (EEMiS), Department of Biology and Environmental Science, Linnaeus University, Kalmar, Sweden

**Keywords:** Virus-host interactions, Bacteriophages, Molecular ecology

## Abstract

Despite important ecological roles posited for virocells (i.e., cells infected with viruses), studying individual cells in situ is technically challenging. We introduce here a novel correlative microscopic approach to study the ecophysiology of virocells. By conducting concerted virusFISH, 16S rRNA FISH, and scanning electron microscopy interrogations of uncultivated archaea, we linked morphologies of various altiarchaeal cells to corresponding phylogenetic signals and indigenous virus infections. While uninfected cells exhibited moderate separation between fluorescence signals of ribosomes and DNA, virocells displayed complete cellular segregation of chromosomal DNA from viral DNA, the latter co-localizing with host ribosome signals. A similar spatial separation was observed in dividing cells, with viral signals congregating near ribosomes at the septum. These observations suggest that replication of these uncultivated viruses occurs alongside host ribosomes, which are used to generate the required proteins for virion assembly. Heavily infected cells sometimes displayed virus-like particles attached to their surface, which agree with virus structures in cells observed via transmission electron microscopy. Consequently, this approach is the first to link genomes of uncultivated viruses to their respective structures and host cells. Our findings shed new light on the complex ecophysiology of archaeal virocells in deep subsurface biofilms and provide a solid framework for future in situ studies of virocells.

Most in situ studies on environmental prokaryotic lytic viruses focus on free virions or their genomes [1]. Recently, the scientific community has begun to investigate ecological roles of the virocells, which are cells that have fallen victim to viral infection and are subject to metabolic conversion [2]. Host molecular machinery is typically reprogrammed rapidly to facilitate virus replication [3], and can even result in distinct cellular compartmentalization for reproduction in the case of highly evolved bacterial jumbo phages [4]. Viruses that infect archaea often possess distinct virion structures, biochemical properties, egress mechanisms, and coordinated virocell takeover motifs (as reviewed in [5–7]). As all of these reports have arisen from isolated archaeal cultures, the scientific community lacks an understanding of the structure and organization of virocells of the uncultivated majority [1].

Recently, advances in fluorescence in situ hybridization (FISH) enabled researchers to detect an uncultivated virus via tagging the viral genome [8, 9] and Schaible et al. to correlated FISH imaging with scanning electron microscopy (SEM) for identification of specific prokaryotes using 16S rRNA tagging [10]. However, the combination of virusFISH (i.e., virus-targeted direct-geneFISH) with SEM has been proposed [11] but not been established, as virusFISH protocols necessitate 300 base pairs double-stranded (ds) probes to enter target cells and thus a harsh temperature treatment of cells, which results in disintegration of the cellular ultrastructure (Supplementary Fig. S1). Here we report a novel correlative microscopic approach to study the ecophysiology of indigenous, uncultivated archaeal virocells by conducting concerted virusFISH, 16S rRNA-based FISH, and SEM analyses. Applied to uncultivated archaea from the deep biosphere, we linked morphologies of various *Candidatus* Altiarchaeum hamiconexum cells to corresponding nucleic acid signals.

Carbon fixing members of the uncultivated archaeal genus *Ca*. Altiarchaeum dominate deep subsurface ecosystems worldwide [12] with uncultivated lytic ds-DNA viruses infecting these archaea and causing profound consequences on carbon cycling in the deep biosphere [8]. The genome of the ds-DNA virus primarily targeting *Ca*. A. hamiconexum and tagged via virusFISH, however, has not been predicted to encode any viral hallmark genes in a previous study [8], which is not surprising since most archaeal viruses encode for novel or previously unknown proteins [5]. By modeling protein tertiary structure from predicted viral genes with AlphaFold [13] and correlating results to annotations of function, we identified a novel auxiliary metabolic gene (i.e., Ni-Fe hydrogenase), a gene encoding a capsid protein, and other virus-associated proteins encoded in the circular viral genome (Supplementary Fig. S2, Supplementary Table S1). Given this in silico evidence confirming the viral nature of the tagged genome, we examined the ultrastructure of *Ca*. A. hamiconexum virocells using a novel correlative microscopic approach.

Conserving cellular ultrastructure is particularly challenging when preparing samples for (virus)FISH and subsequent SEM. A distinct characteristic of *Ca*. A. hamiconexum are their extracellular hami structures, which are extracellular cell surface appendages with a basal barbwire structure and nano-grappling terminal hooks to interconnect cells [14]. By carefully balancing fixation reagent concentrations with critical point drying (for details please see Supplementary Methods, Supplementary Fig. S1 illustrates insufficient sample preservation for comparison), we successfully preserved the hami, and thus ultrastructure of *Ca*. A. hamiconexum cells throughout virusFISH preparation (Fig. 1). Using gridded coverslips to ensure proper correlation of fluorescence and SEM signals (Fig. 1a, Supplementary Fig. S3), spatial separation between ribosomes and chromosomal DNA was apparent in most uninfected cells. Although this phenomenon has previously been observed for archaea larger than altiarchaeal cells (i.e., Asgardarchaeota) [15], we observe a comparable segregation in ribocells (cells showing no infection) of small Altiarchaea of the DPANN superphylum (700–800 nm diameter). A similar segregation was observed in cells undergoing division, with DNA localizing about the outer poles of each of the daughter cells. At the same time, our approach enabled the infrequent detection of small DNA-filled vesicles (diameter: 250–300 nm, *n* = 3) attached to *Ca*. A. hamiconexum ribocells (Fig. 1b). We hypothesize that these vesicles play an important role in inter-kingdom lateral gene transfer, which has recently been reported in *Ca*. Altiarchaea [16].

**Fig. 1:**
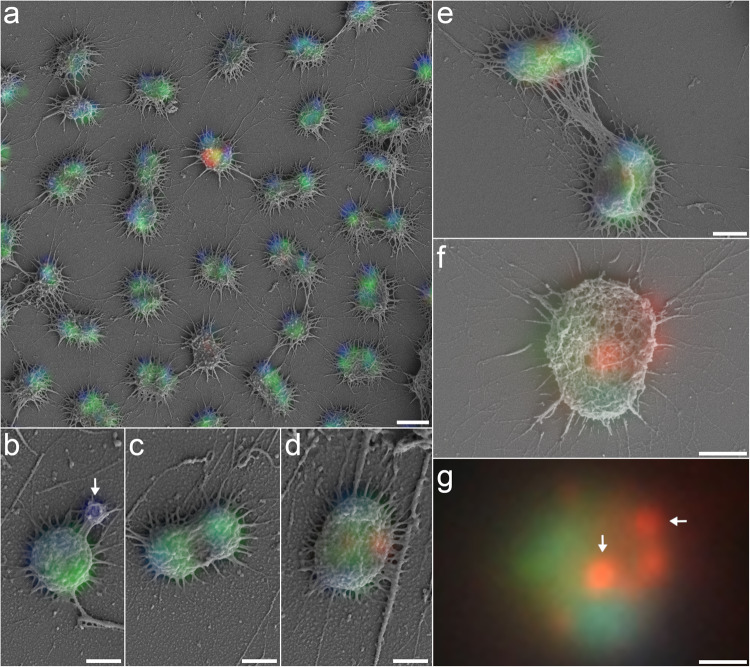
Overlays of fluorescence and scanning electron micrographs of naturally occurring *Ca.* A. hamiconexum biofilms. Blue fluorescence signal corresponds to DAPI, green to the SMARCH714 probe [20] labeling the 16S rRNA of *Ca.* A. hamiconexum (Atto488) and red the virusFISH probes [8] labeling the viral genome (Alexa594); **a** Overview of single infected cell between multiple non-infected cells demonstrating the successful correlation of the two imaging techniques due to sample preservation. **b** Uninfected single cell. Arrow points at a putative vesicle. **c** Uninfected dividing cells. **d** Infected swollen cell. **e** Dividing infected cells. **f** Single infected cell. **g** Overlay of the fluorescence images of **f**. Arrows point at spherical viral signals. Scale bars: 1 µm (**a**), 500 nm (**b**–**g**). We provide a color-blind friendly image of this figure as Supplementary Fig. S4; single channel images are provided as Supplementary Figs. S5–S8.

Altiarchaeal cells infected with uncultivated viruses (infection rate 5% [11]) were 10–20% larger and slightly more spherical than their ribocell counterparts (Fig. 2b; 30 infected and 145 non-infected cells; Wilcoxon test, two-sided, *p* value < 0.001; Supplementary Table S2, Supplementary Figs. S9, S10). Virocell enlargement has been reported previously in pure culture, with infected *Sulfolobus islandicus* cells increasing their size by several orders of magnitude [6]. In dividing *Ca*. A. hamiconexum cells, virus signals accumulated along the cell division septum, indicating that viral reproduction occurs in the cytosol between the two daughter cells (Fig. 1d, e). This spatial separation of chromosomal DNA, ribosomes, and virion synthesis was also observed in individual cocci (Fig. 1f, g). Upon comparing these observations with ultrathin sections of *Ca*. A. hamiconexum cells, we confirmed a distinct segregation of viral reproduction activities from other cytosolic spaces (Fig. 2c, Supplementary Fig. S12). An accumulation of virus signals arranged in sphere-like intracellular structures observed via fluorescence microscopy suggested an organized packing of virions (Figs. 1g and 2a). However, virocells did not exhibit these sphere-like structures in most instances, but rather a dense agglomeration of virus signals (e.g., virus signals in Fig. 2a). These observations most likely reflect different stages of the viral reproduction cycle, yet all of these are spatially separated from host chromosomes.

**Fig. 2:**
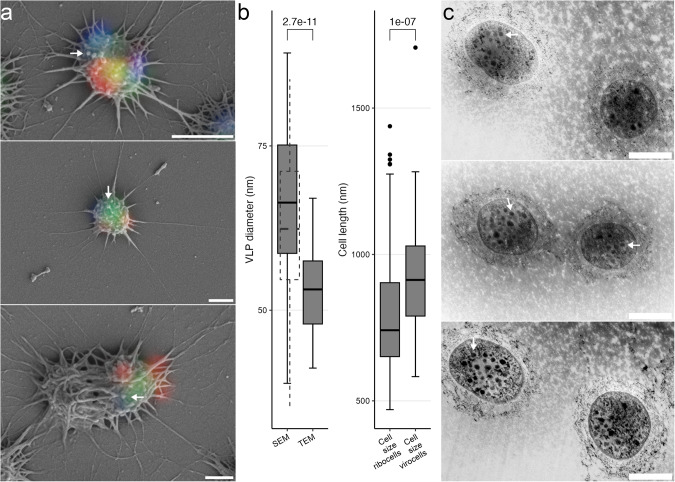
Identification of intracellular and putative extracellular viral particles. **a** Overlays of fluorescence and scanning electron micrographs (sampled in 2022). Arrows point at putative extracellular VLPs. Blue fluorescence signal corresponds to DAPI, green to the SMARCH714 probe [20] labeling the 16S rRNA of *Ca*. A. hamiconexum (Atto488) and red the virusFISH probes [8] labeling the viral genome (Alexa594). **b** Boxplot indicating the measured size of the VLPs measured in SEM and TEM (average diameter of VLPs in TEM 53 nm (*n* *=* 56) vs. SEM 65 nm (*n* *=* 71) t-test *p* value *=* 2.7·10^−11^) The dashed boxplot within the SEM column represents the hypothetical data after subtraction of the 4 nm Pt/Pd layer (significance of t-test above boxplots). In comparison to the measured archaeal cell size (30 infected and 145 non-infected cells; Wilcoxon test, *p* value *<* 0.001) (significance of Wilcoxon test above boxplots). **c** TEM images (sampled in 2018 [8]). Arrows point at putative VLPs within *Ca*. A. hamiconexum cells. Scale bars 500 nm.

Strong virus signals not segregating into individual spheres often co-occurred with changes to the host cell’s surface structure. These alterations manifested as small virus-like particles (VLPs) attached to the surfaces of *Ca*. A. hamiconexum cells (Fig. 2a). While the virion architecture of these uncultivated viruses remains unknown, these structures agree with transmission electron microscopy (TEM) images herein and elsewhere [8, 17]. We compared the size of intracellular putative VLPs of TEM thin sections and the extracellular VLPs identified via correlative microscopy finding a significant difference in size [average diameter of VLPs in TEM 53 nm (*n* = 56) vs. SEM 65 nm (*n* = 71) t-test *p* value = 2.7·10^−11^; Supplementary Table S3, Fig. 2, Supplementary Figs. S11, S12]. Although the difference in size might be due to the application of different imaging techniques or the different states of virus maturation (e.g., intracellular and extracellular, presence of the capsids), the sizes of the VLPs are in the expected range of archaeal viruses [18]. However, the correlative microscopic method described here links metagenomic data to the structure of uncultivated viruses, and thus enabled elucidating morphological traits of viruses whose existence is known only from sequence data. We suggest that this novel approach could be easily adapted to other sample matrices than subsurface biofilms, which per se represent a challenge due to the complexity of the biofilm matrix.

Microbes of the deep biosphere remain some of the most enigmatic biological entities on Earth due to limitation in sampling opportunities of the deep subsurface. While "omics" approaches are mostly used to decipher microbial traits in the deep subsurface [19], advances at single-cell level to understand ecophysiology of these organisms and associated viral infections remain the exception. Our novel protocol enabling the ability to spot rare infection events of individual cells presents a major step forward in understanding the little characterized deep biosphere at individual virocell level and demonstrates specific morphologies from vesicles to size alteration of uncultivated archaea. The spatial-functional distribution within virocells reveals a high degree of sub-cellular organization in deep-branching archaea, which might be required for the viruses to replicate and warrants future investigation necessitating sophisticated method development tied to high-resolution imaging, like TEM [9]. While the molecular mechanisms underlying such virus replication in deep-branching archaea remain unknown, we suggest that sub-cellular organizations in ribocells and virocells play an important role in microbial physiology also outside well-characterized model systems and might encompass the entire tree of archaea.

## Supplementary information

Main Supplementary Information

Supplementary Information 2

Supplementary Information 3

Table S1

Table S2 and S3
