# Supplementary material for: Spatio-functional organization in virocells of small uncultivated archaea from the deep biosphere
Source: ISME J. 2023 Jul 19;17(10):1789–92. doi: 10.1038/s41396-023-01474-1 (PMC10504349; doi:10.1038/s41396-023-01474-1)
Supplement: Supplementary file 1 — Main Supplementary Information [file 41396_2023_1474_MOESM1_ESM.docx]

Supplementary Information for

**Spatio-functional organization in virocells of small uncultivated archaea from the deep biosphere**

Indra Banas^1,2^, Sarah P. Esser^1,2^, Victoria Turzynski^1,2^, André Soares^1,2^, Polina Novikova^3^, Patrick May^3^, Cristina Moraru^1,4^, Mike Hasenberg^5^, Janina Rahlff^2,#^, Paul Wilmes^3,6^, Andreas Klingl^7^*, and Alexander J. Probst^1,2,8,9^*

^1^ Environmental Metagenomics, Research Center One Health Ruhr of the University Alliance Ruhr, Faculty of Chemistry, University of Duisburg-Essen

^2^ Group for Aquatic Microbial Ecology, Environmental Microbiology and Biotechnology University Duisburg-Essen, Essen, Germany

^3^Luxembourg Centre for Systems Biomedicine, University of Luxembourg, Esch-sur-Alzette, Luxembourg
^4^Institute for Chemistry and Biology of the Marine Environment (ICBM), Carl-von-Ossietzky-University Oldenburg, Oldenburg, Germany

^5^Imaging Center Essen, EMU, Essen, Germany

^#^Present address: Centre for Ecology and Evolution in Microbial Model Systems (EEMiS), Department of Biology and Environmental Science, Linnaeus University, Kalmar, Sweden

^6^ Department of Life Sciences and Medicine, Faculty of Science, Technology and Medicine, University of Luxembourg, Belvaux, Luxembourg
^7^ Plant Development & Electron Microscopy, Biocenter LMU Munich, Planegg-Martinsried, Germany

^8^ Centre of Water and Environmental Research (ZWU), University of Duisburg-Essen, Essen, Germany

^9^ Center of Medical Biotechnology (ZMB), University of Duisburg-Essen, Essen, Germany

*to whom the correspondence should be addressed: andreas.klingl@biologie.uni-muenchen.de and alexander.probst@uni-due.de

*List of content:*

1. *Supplementary Materials and Methods*
2. *Supplementary Table*
3. *Supplementary Figures*
4. *References*
5. **Materials and Methods**

***Annotation of the viral genome.*** The previously-published 8.9 kb viral genome Altivir_1_MSI_BF_2012 (*GenBank accession number of the genome: MW522970.1*) (1) was used to predict 13 viral open reading frames (prodigal version2.6.3) (2). Sequences of viral proteins were subject to annotation using three protein structure prediction methods: AlphaFold2 (3), ProFunc (4), and trRosetta (5). Protein structures were predicted with AlphaFold2 using either of two databases for multiple sequence alignment (MSA) generation - MMseqs2 (6) or Jackhmmer (7). The model built with higher MSA coverage was further used for the structure annotation with the ProFunc web server. ProFunc report provided *Sequence search vs existing Protein Data Bank (PDB)* entries and *3D functional template search* sections which were used for structure-based protein annotation. Matches with confidence scores [certain matches (E-value <10-6), probable matches (10-6 < E-value <0.01), possible matches (0.01 < E-value < 0.1)] were considered for the resulting annotation. Additionally, trRosetta was used as a second structure prediction method. It provided hits for structures with detectable homologous matches in PDB (https://www.rcsb.org/). Candidate molecules reported in available templates with the highest identity and coverage were used for protein structure annotation. By default, trRosetta used a template for prediction and provides a match in PDB only if it satisfies the following condition for the predicted model: confidence > 0.6, E-value < 0.001 and coverage > 0.3. The respective AlphaFold protein foldings were visualized with Geneious prime (version 11.1) (8) and FirstGlance in Jmol (https://proteopedia.org/wiki/fgij/, accessed Sep. 2022).

***Sampling and sample fixation.*** Biofilm flocks were collected in February 2022 from the cold sulfidic spring Muehlbacher Schwefelquelle, Isling (MSI) in Regensburg, Germany (N 48° 59.142, E 012° 07.636) as described previously (9). Biofilm flocks were fixed in the field for 1 h by using 16 % methanol-free formaldehyde (Thermo Scientific, IL, USA) resulting in a final concentration of 3 % (v/v). Afterwards, the samples were washed three times using 75 mM cacodylic acid sodium salt 3-hydrate *BioChemica* (Cacodylate buffer, AppliChem, Germany, 2 mM MgCl_2_, pH 6.5-7) and a dehydration series was performed by incubation for 10 min at 30 %, 50 % and 70 % ethanol. The supernatant was exchanged every step after 10 min centrifugation at 4 °C and 10,000 g (equivalent 21130 rcf). The samples were stored in absolute ethanol at -20 °C until further processing.

***Virus-targeted direct-geneFISH (virusFISH)*** was performed with the same probe sequences as described previously with the following minor changes (1): The flocks were deposited on an ibidi µ-Dish 35 mm, high Grid-500 Glass Bottom (ibidi GmbH, 82166 Gräfelfing, Germany, www.ibidi.com). Biofilms were evenly spread on the grid. Once the ethanol had almost completely evaporated and the cells had adhered to the surface, the grid was covered with 90 µL of a 20 % formamide hybridization buffer, with a gene probe concentration of 30 pg/µL for eleven polynucleotides (330 pg/µL in total) and rRNA probe concentration of 1 pmol/µl. After denaturation at 85 °C for 30 min, hybridization was performed at 46 °C for 3 h. The sample was washed in the preheated buffer (suitable for a hybridization buffer with 20 % formamide) for 15 min. Subsequently, the specimen was transferred in 1X Phosphate Buffered Saline (PBS, Sigma Aldrich, Germany, pH 7.4) for 20 min with the dish being merged in an excess volume buffer (about 100 mL). Afterwards the dish was filled with ice cold deionized water followed by ethanol. Prior to drying, the grid was covered with a 4′,6-diamidin-2-phenylindole (DAPI, Thermo Fisher Scientific, Waltham, MA, USA, 4 μg/mL) solution for three min and washed with deionized water. The sample was stored in 75 mM cacodylate buffer (2 mM MgCl_2_, pH 6.5-7) at 4 °C until imaging. See step by step protocol "Supplementary Information 2.pdf"

***Scanning electron microscopy (SEM) sample preparation.*** After fluorescence microscopy (FM) imaging, the sample was incubated with 2.5 % glutardialdehyde (Carl Roth, Germany) for 30 min and stored in 75 mM cacodylate buffer (AppliChem, Germany , 2 mM MgCl_2_, pH 6.5-7) at 4 °C overnight. For SEM sample preparation, the sample was washed in cacodylate buffer, incubated 30 min in 1 % osmium tetroxide (Polysciences, Inc., PA, USA) on ice and washed again with cacodylate buffer followed by deionized water. Prior to the acetone dehydration series (10 %, 20 %, 40 %, 60 %, 80 %, 100 %, and anhydrous acetone) the grided glass bottom was carefully removed from the plastic dish using a diamond pen. The sample was kept in fresh anhydrous acetone overnight in the fridge, then critical point dried (Polaron - Range CPD7501) and coated with a 2 nm platin/palladium layer (Leica EM ACE 600 Sputter Coater). The sample was stored at room temperature under vacuum until imaging. See step by step protocol "Supplementary Information 3.pdf"

**Transmission electron microscopy (TEM) ultrathin sections** Biofilm flocks were fixed, processed and imaged as described previously (1).

***Imaging.*** FM imaging was performed with an Axio Imager M2m epifluorescence microscope (X-Cite XYLIS Broad Spectrum LED Illumination System, Excelitas) equipped with an AxioCam MRm and a Zen 3.4 Pro software or a Zeiss LSM710 confocal microscope (561 nm laser, 488 nm Argon Multiline and 405 nm Diode laser) with spectral detection and ZEN system 2012 (black Edition; both Carl Zeiss Microscopy GmbH, Jena, Germany).

In both systems, flocks were first located using a 20x objective and noting the coordinates according to the grid system. Then an oil immersion objective was used for high resolution (100x at Axio Imager M2m/ 63x plan-Apochromat at Zeiss LSM710).

SEM imaging was performed using a ZEISS- Crossbeam 540 and Secondary electron detector. Scan rotation was used for rotating the specimen in a similar orientation as during FM image acquisition, which was performed in high resolution column mode with a probe current of 38 pA and a landing energy of 1.5 kV. For localization and correlation, first overview micrographs of whole biofilms were taken, followed by increasing magnification down to micrographs of single cells.

***Data processing.*** FM micrographs were created using Zeiss Zen software (Zen 3.4 Pro software, blue Edition ZEN system 2012, black Edition) as czi files and exported as jpg of single channel images and overlays. Scanning electron micrographs were saved as tiff files. Overlays were created using GIMP 2.10. The SEM micrographs were kept in original size while the FM images were scaled by calculating the scale bar pixel sizes and slightly rotated if necessary. For size measurement of archaeal cells and VLPs ImageJ (Version 2.0.0-rc-69/1.52p) was used.

**Data availability**. All imaging data is available on FigShare under https://figshare.com/projects/Spatio-functional_organization_in_virocells_of_small_uncultivated_archaea_from_the_deep_biosphere/162052.

1. **Supplementary Tables**

**Table S1** | Structural annotation of identified viral proteins *(GenBank accession number of the genome: MW522970.1*). This table is provided as a separate file <structure_annotation_table.xlsx>.

**Table S2 |** Summary of cell size measurements of *Ca.* A. hamiconexum. See measured cells in Figure S10.

|  | width (nm) | | | length (nm) | | |
| --- | --- | --- | --- | --- | --- | --- |
|  | ribocell | virocell | difference | ribocell | virocell | difference |
| Mean | 723.9 | 824.9 | 101.0 (113%) | 849.7 | 1023.5 | 173.7 (120%) |
| Standarddeviation | 120.6 | 124.0 |  | 210.1 | 197.1 |  |
| Median | 704.1 | 803.4 | 99.3 (114%) | 835.4 | 1017.3 | 181.9 (121%) |

**Table S3 |** Summary of VLP size measurements of *Ca.* A. hamiconexum via SEM and TEM. See measured particles in Figure S11 and S12. Please note that VLPs detected via SEM were extracellular, while VLPs detected via TEM were intracellular.

|  | length measured (nm) | |
| --- | --- | --- |
|  | via SEM | via TEM |
| VLPs measured | 72 | 56 |
| Mean | 65.65 | 53.24 |
| Standard deviation | 12.12 | 6.16 |
| Median | 66.37 | 53.17 |

1. **Supplementary Figures**


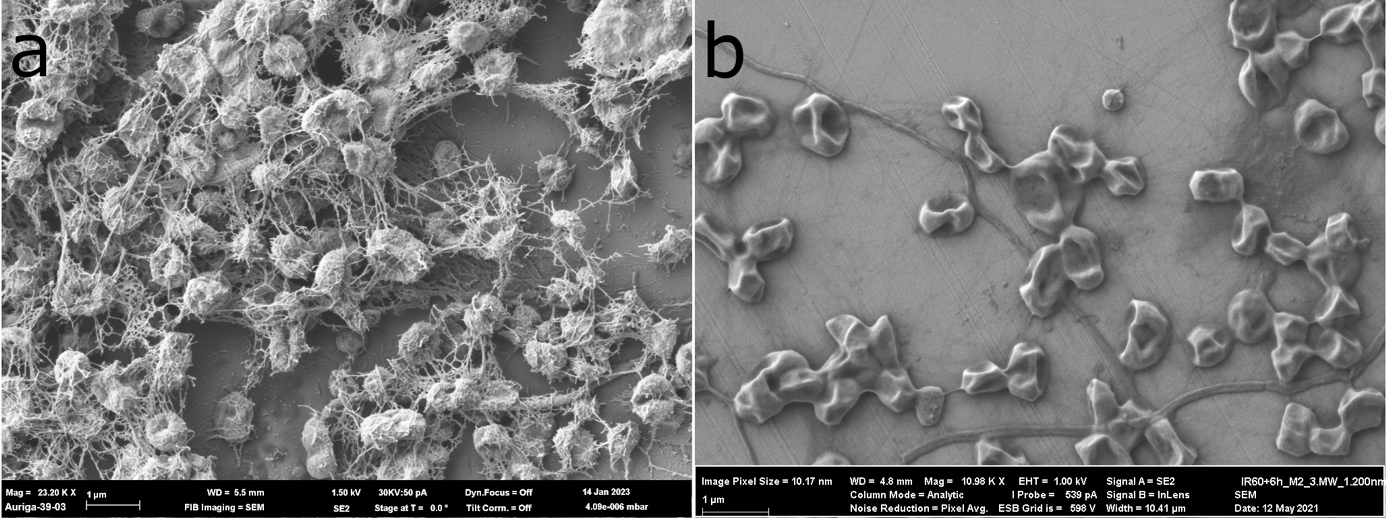


**Figure S1** | Impact of sample preparation on cellular ultrastructure of Ca. A. hamiconexum in scanning electron micrographs; a: Cells directly prepared for SEM imaging, which shows the conserved ultrastructure including hami (cell surface appendages). b: Cells prepared for virusFISH, without establishment of the virusFISH SEM sample preparation protocol, resulting in artifacts regarding cell morphology and loss of ultrastructure information (e.g., hami are no longer visible).


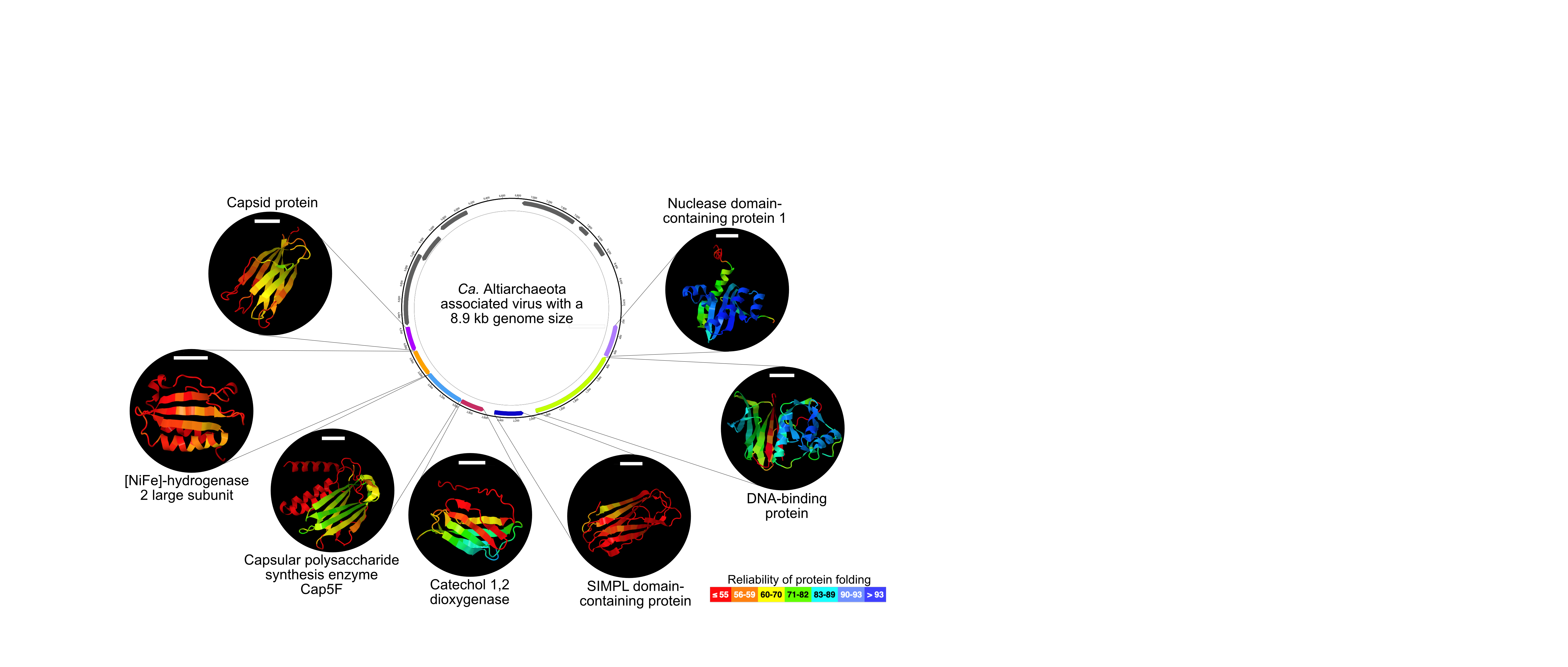


**Figure S2** | *Ca.* A. hamiconexum virus with seven visualized protein tertiary structures (FristGlance in Jmol; https://proteopedia.org/wiki/fgij/). The color of the predicted protein structure is dependent on the reliability of the predicted folding, with red being the lowest and dark blue being the highest reliability. Grey predicted proteins at the viral genome could not be structurally predicted by AlphaFold. Scale bar: 10 Å.

**
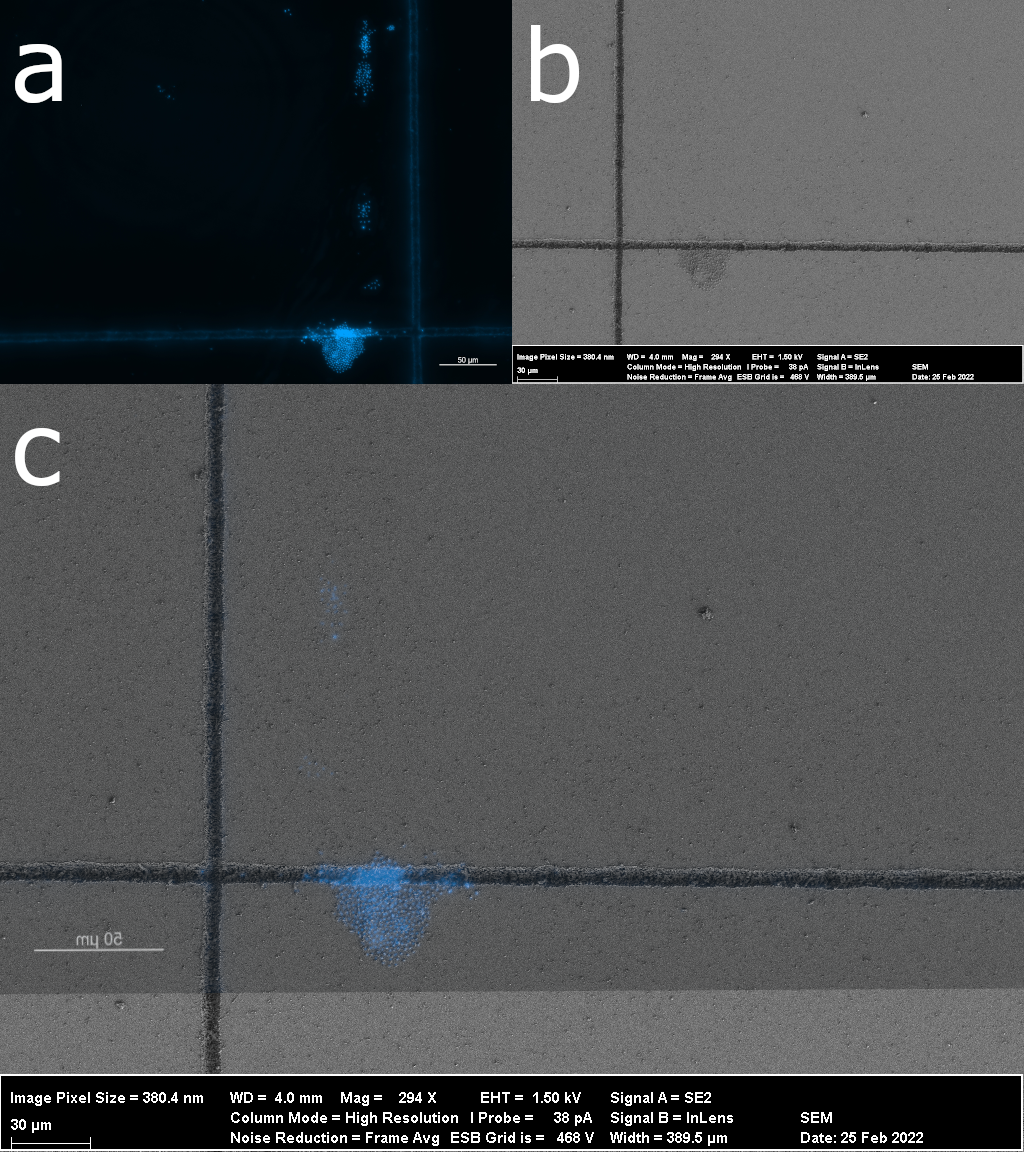
**

**Figure S3** | Example of localization and correlation of a biofilm flock using the gridded coverslip. a: Original fluorescence micrograph (DAPI channel, scale bar 50 µm). b: Original scanning electron micrograph (scale bar 30 µm). c: Overlay of a scanning electron and fluorescence micrograph overview (FM was mirrored and scaled).


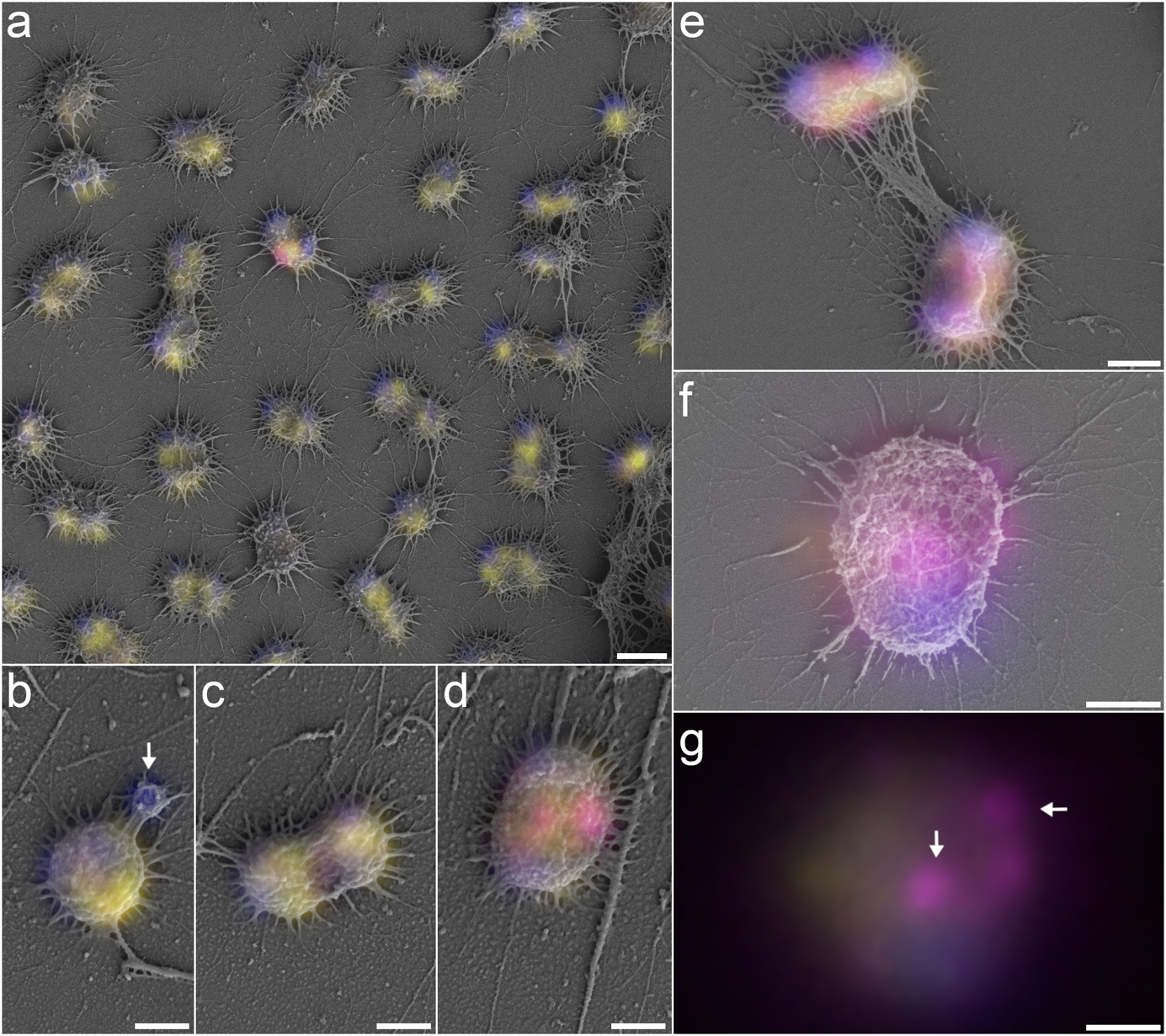


**Figure S 4** | Colorblind friendly version of Figure 1. Overlays of fluorescence and scanning electron micrographs of naturally occurring Ca. A. hamiconexum biofilms. Blue fluorescence signal corresponds to DAPI, yellow to the SMARCH714 probe labeling the 16S rRNA of Ca. A. hamiconexum (Atto 488) and magenta the virusFISH probes (1) labeling the viral genome (10) (Alexa594); a: overview of single infected cell between multiple non infected cells demonstrating the successful correlation of the two imaging techniques due to sample preservation. b: uninfected single cell. Arrow points at a putative vesicle. c: uninfected dividing cells. d: infected swollen cell. e: dividing infected cells. f: single infected cell. g: overlay of the fluorescence images of f. Arrows point at spherical viral signals. Scale bars: 1 µm (a), 500 nm (b-g.)


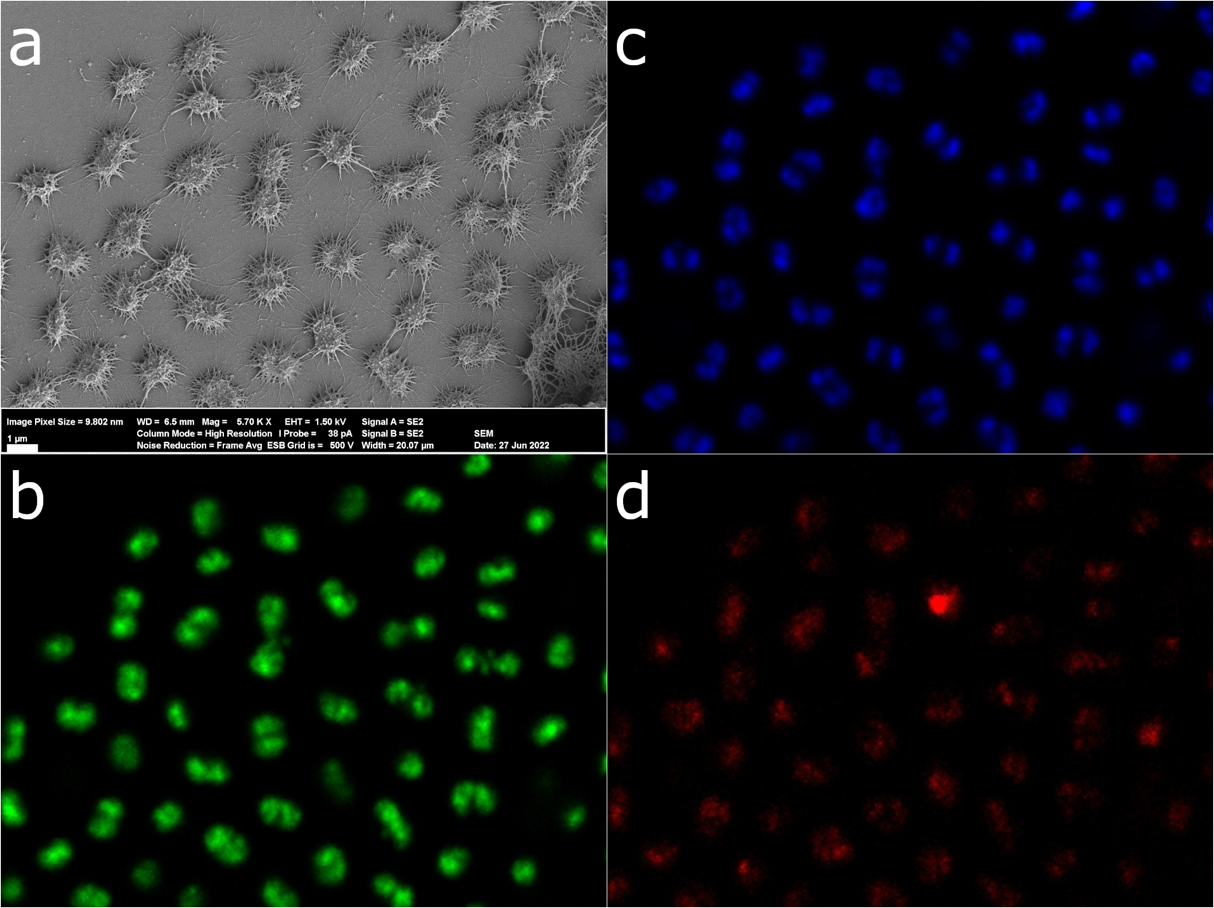
 **Figure S5** | Single channel images of Figure 1a. a: Original scanning electron micrograph. b-d: fluorescence micrograph single channels scaled to panel a. b: SMARCH714 probe (10) labeling the 16S rRNA of Ca. Altiarchaeum. c: DAPI signal. d: virusFISH probes (1) labeling the viral genome. Scale bar 1 µm valid for all images


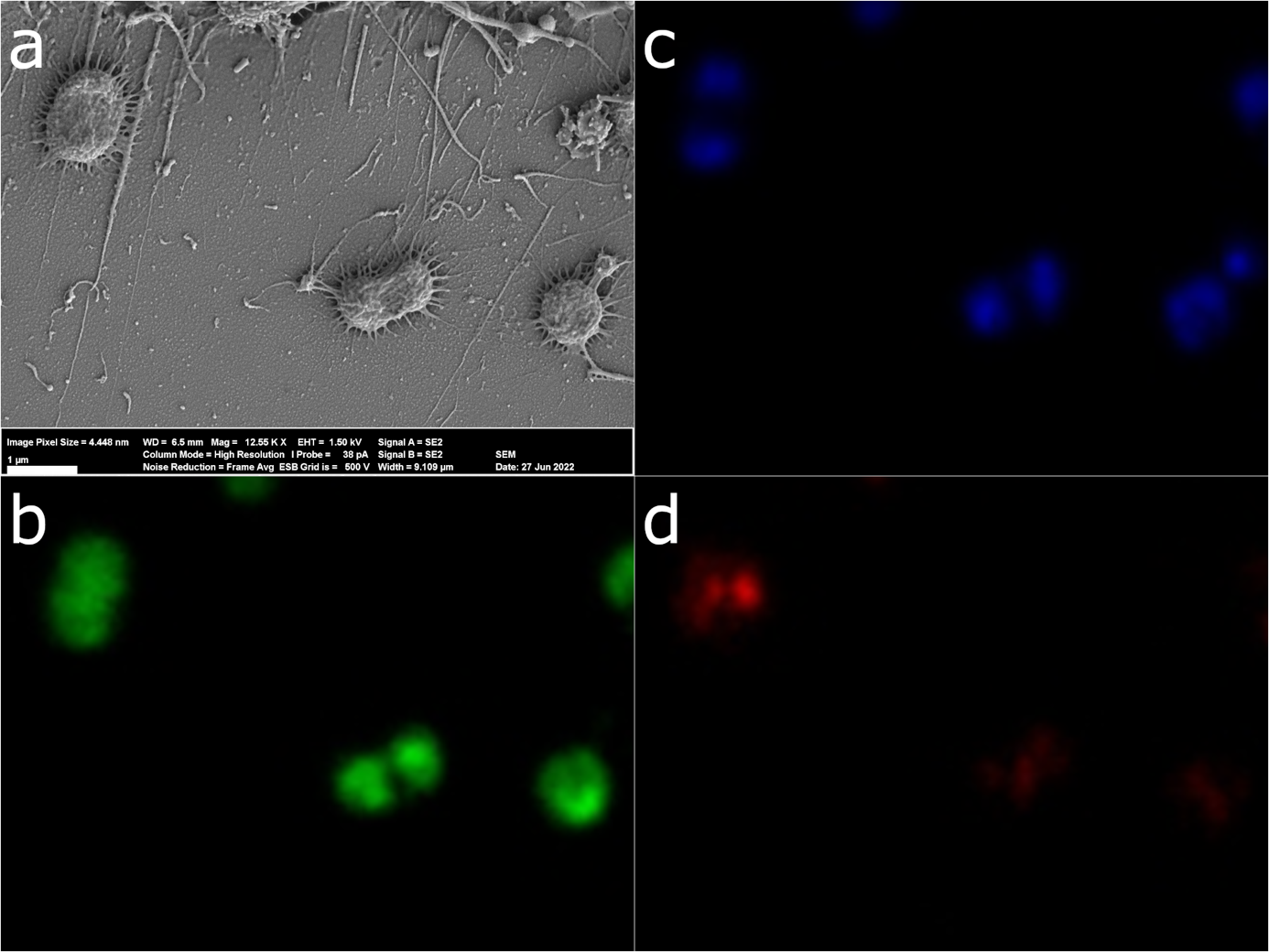
**Figure S6** | Single channel images of Figure 1b-d. a: Original scanning electron micrograph. b-d: fluorescence micrograph single channels scaled to panel a. b: SMARCH714 probe (10) labeling the 16S rRNA of Ca. Altiarchaeum. c: DAPI signal. d: virusFISH probes (1) labeling the viral genome. Scale bar 1 µm valid for all images.


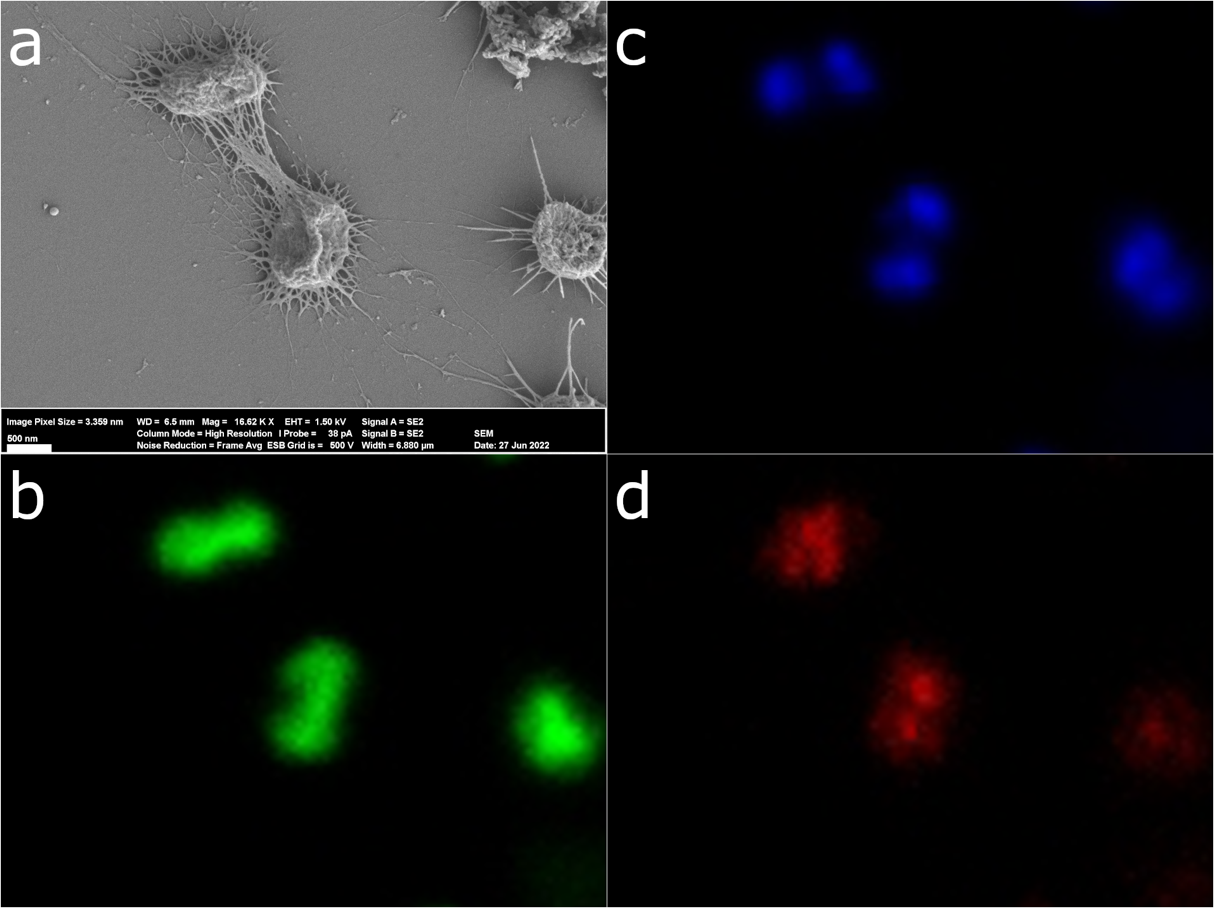


**Figure S7** | Single channel images of Figure 1e. a: Original scanning electron micrograph. b-d: fluorescence micrograph single channels scaled to panel a. b: SMARCH714 probe (10) labeling the 16S rRNA of Ca. Altiarchaeum. c: DAPI signal. d: virusFISH probes (1) labeling the viral genome. Scale bar 1 µm valid for all images.


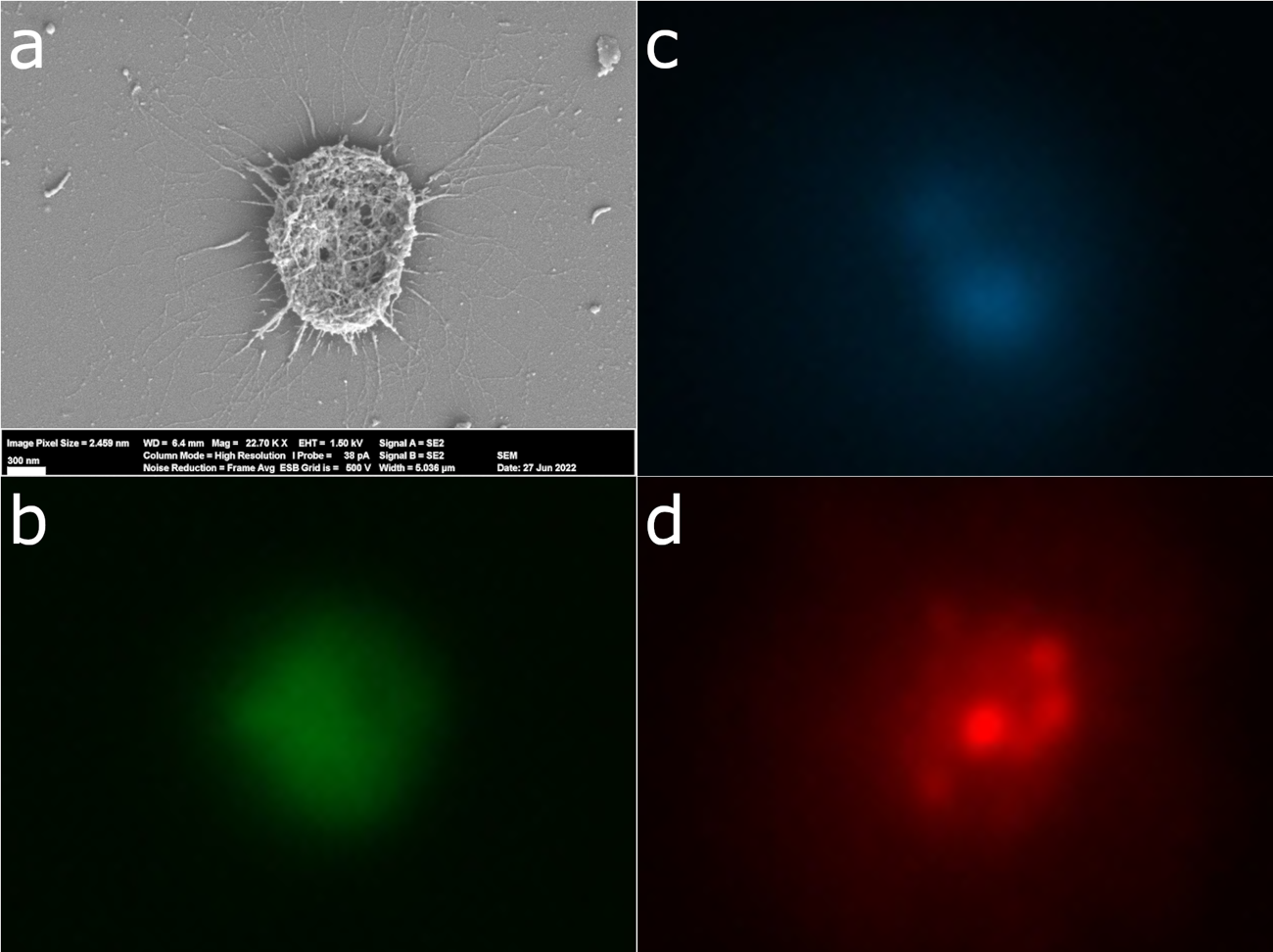
**Figure S8** | Single channel images of Figure 1e and f. a: Original scanning electron micrograph. b-d: fluorescence micrograph single channels scaled to panel a. b: SMARCH714 probe (10) labeling the 16S rRNA of Ca. Altiarchaeum. c: DAPI signal. d: virusFISH probes (1) labeling the viral genome. Scale bar 1 µm valid for all images.


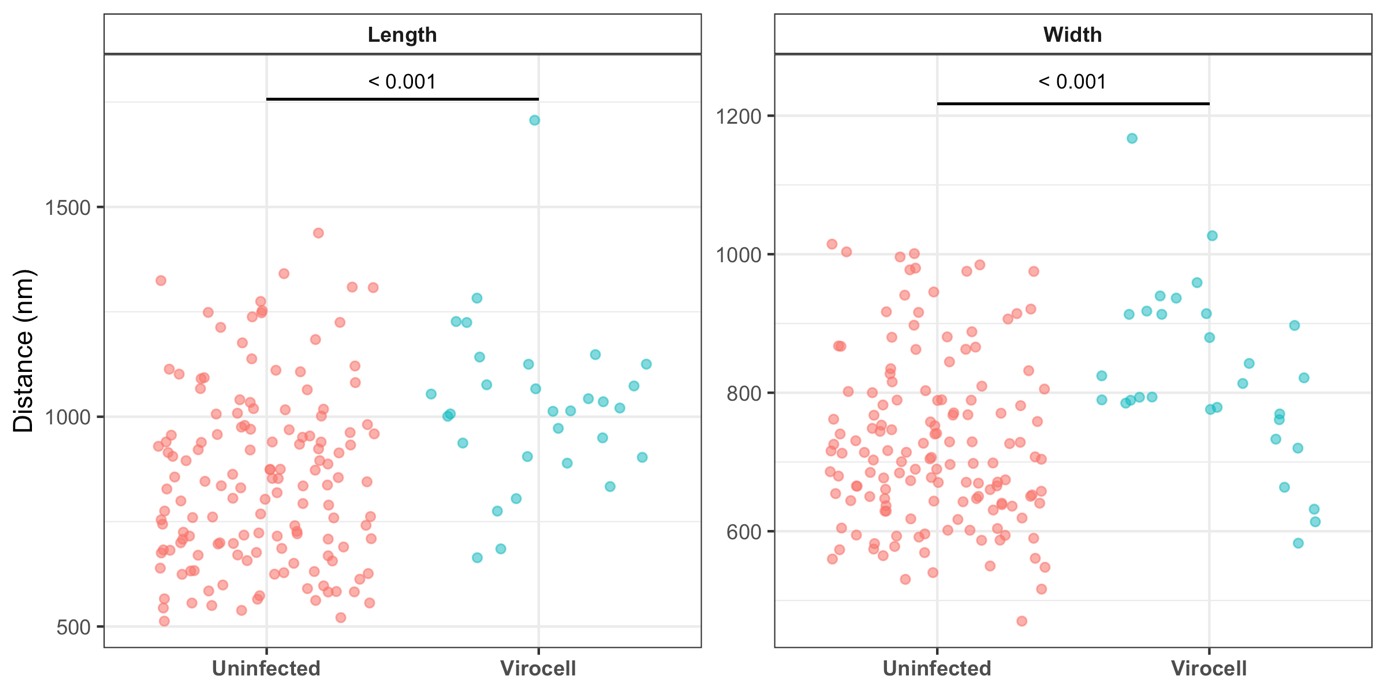


**Figure S9** | Size distribution of all measured archaeal cells. Significance is determined by a Wilcoxon test (<0.001 indicates the p-value).


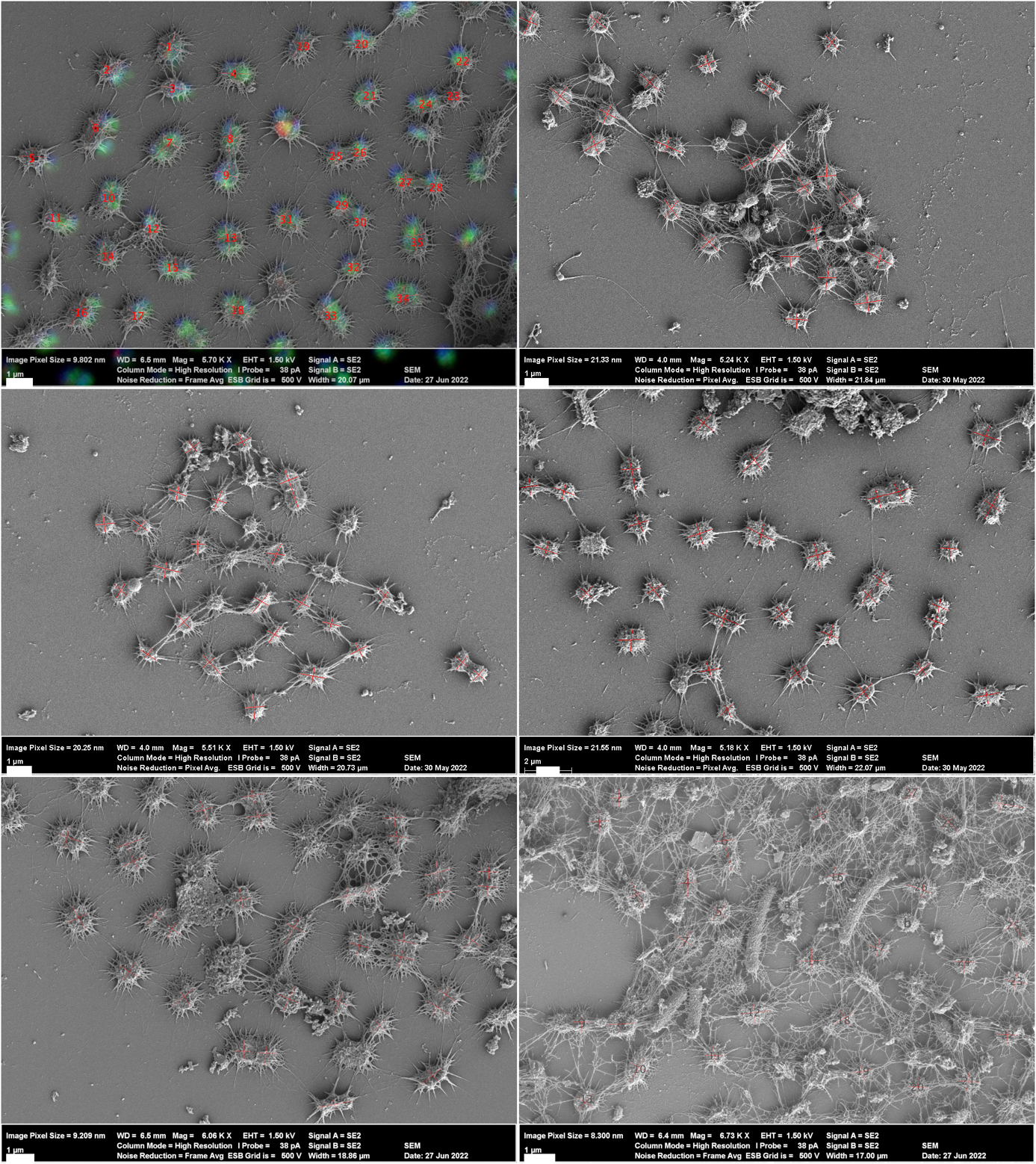


**Figure S10** | Fluorescence microscopy/SEM overlay (top left) and SEM images (other panels) of biofilm cells which were used for size comparison. Red lines indicate the measured length calculated based on the pixel size. The fluorescence image was used to differentiate dividing cells (split DAPI signal; compare main Figure 1d), separated cells (split DAPI and 16S rRNA gene FISH signal; compare main Figure 1c), and infected cells (singal in virusFISH; compare main Figure 1f). Some cells were not measured because they had no 16S rRNA signal (all scale bars 1 µm). For full size images, please see Figshare https://figshare.com/projects/Spatio-functional_organization_in_virocells_of_small_uncultivated_archaea_from_the_deep_biosphere/162052.


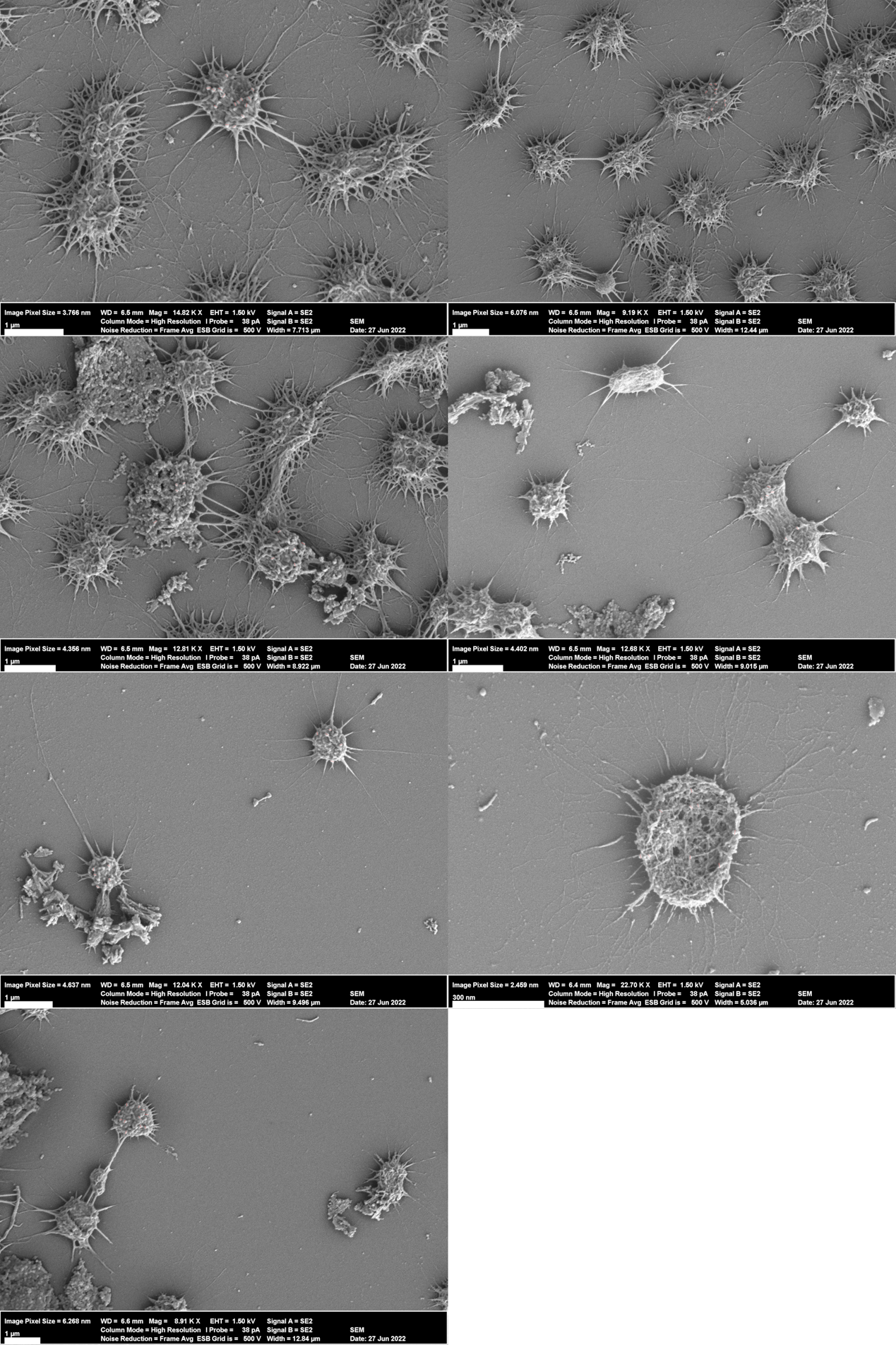


Figure S 11 | SEM of Ca. A. hamiconexum cells with VLPs, which were used for size comparison. Red lines indicate the measured length calculated based on the pixel size (scale bars 1 µm). For full size images, please see Figshare https://figshare.com/projects/Spatio-functional_organization_in_virocells_of_small_uncultivated_archaea_from_the_deep_biosphere/162052.


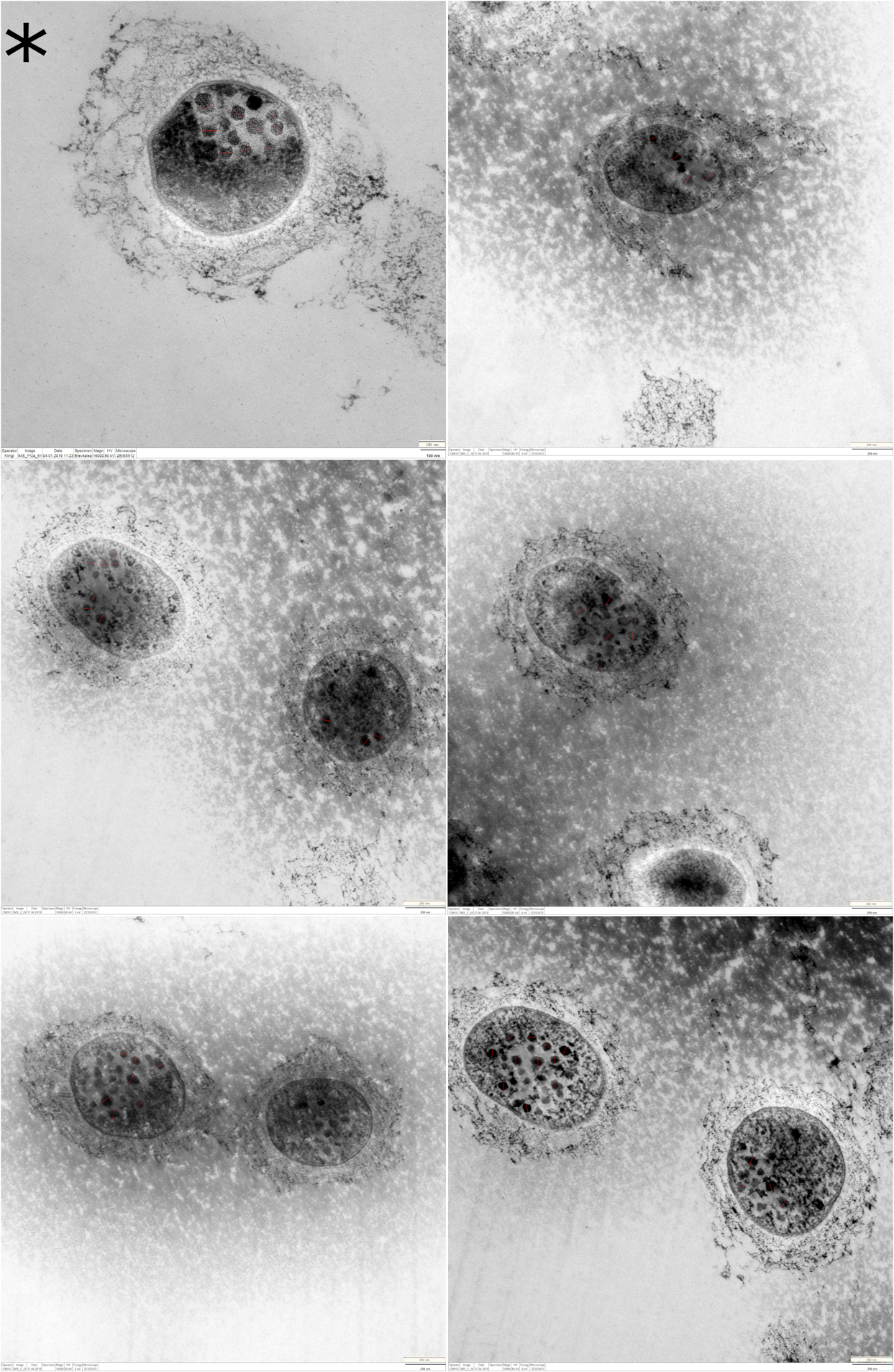


**Figure S 12** | TEM of Ca. A. hamiconexum cells with VLPs, which were used for size comparison. Red lines indicate the measured length calculated based on the pixel size (scale bars 250 nm). *Image taken from Rahlff et al. 2021 (1), scale bar 100 nm. For full size images, please see Figshare https://figshare.com/projects/Spatio-functional_organization_in_virocells_of_small_uncultivated_archaea_from_the_deep_biosphere/162052.

1. **References**

1. Rahlff J, Turzynski V, Esser SP, Monsees I, Bornemann TLV, Figueroa-Gonzalez PA, et al. Lytic archaeal viruses infect abundant primary producers in Earth’s crust. Nat Commun. 2021 Jul 30;12(1):4642.

2. Hyatt D, Chen GL, LoCascio PF, Land ML, Larimer FW, Hauser LJ. Prodigal: prokaryotic gene recognition and translation initiation site identification. BMC Bioinformatics. 2010 Mar 8;11(1):119.

3. Mirdita M, Schütze K, Moriwaki Y, Heo L, Ovchinnikov S, Steinegger M. ColabFold: making protein folding accessible to all. Nat Methods. 2022 Jun;19(6):679–82.

4. Laskowski RA, Watson JD, Thornton JM. ProFunc: a server for predicting protein function from 3D structure. Nucleic Acids Research. 2005 Jul 1;33(suppl_2):W89–93.

5. Du Z, Su H, Wang W, Ye L, Wei H, Peng Z, et al. The trRosetta server for fast and accurate protein structure prediction. Nat Protoc. 2021 Dec;16(12):5634–51.

6. Steinegger M, Söding J. MMseqs2 enables sensitive protein sequence searching for the analysis of massive data sets. Nat Biotechnol. 2017 Nov;35(11):1026–8.

7. Potter SC, Luciani A, Eddy SR, Park Y, Lopez R, Finn RD. HMMER web server: 2018 update. Nucleic Acids Research. 2018 Jul 2;46(W1):W200–4.

8. Kearse M, Moir R, Wilson A, Stones-Havas S, Cheung M, Sturrock S, et al. Geneious Basic: An integrated and extendable desktop software platform for the organization and analysis of sequence data. Bioinformatics. 2012 Jun 15;28(12):1647–9.

9. Probst AJ, Weinmaier T, Raymann K, Perras A, Emerson JB, Rattei T, et al. Biology of a widespread uncultivated archaeon that contributes to carbon fixation in the subsurface. Nat Commun. 2014 online;5:5497.

10. Moissl C, Rudolph C, Rachel R, Koch M, Huber R. In situ growth of the novel SM1 euryarchaeon from a string-of-pearls-like microbial community in its cold biotope, its physical separation and insights into its structure and physiology. Arch Microbiol. 2003 Sep 1;180(3):211–7.
