# Supplementary material for: Spatio-functional organization in virocells of small uncultivated archaea from the deep biosphere
Source: ISME J. 2023 Jul 19;17(10):1789–92. doi: 10.1038/s41396-023-01474-1 (PMC10504349; doi:10.1038/s41396-023-01474-1)
Supplement: Supplementary file 2 — Supplementary Information 2 [file 41396_2023_1474_MOESM2_ESM.pdf]

VirusFISH on ibidi-dish

Date: 2023-02-22
Tags: virusFISH direct-geneFISH DGF Altiarchaeum
Created by: Indra Banas

This workflow is the first part of the CLEM sample preparation

Implement the virusFISH workflow first, establish the correct FA concentration for the probes, and optimise washing steps based on negative control

For details on probe design, preparations of probe stock solutions, hybridisation buffer and implementation of negative controls, please read the original publications:

Direct-geneFISH: Barrero-Canosa J, Moraru C, Zeugner L, Fuchs BM, Amann R. Direct-geneFISH: a simplified protocol for the simultaneous detection and quantification of genes and rRNA in microorganisms. Environmental Microbiology. 2017;19(1):70–82.

VirusFISH: Rahlff J, Turzynski V, Esser SP, Monsees I, Bornemann TLV, Figueroa-Gonzalez PA, et al. Lytic archaeal viruses infect abundant primary producers in Earth's crust. Nat Commun. 2021 Jul 30;12(1):4642

Background:

Direct-geneFISH allows for simultaneous gene and 16S rRNA hybridisation within ~6 h.16S rRNA is targeted by an oligonucleotide probe carrying 1-4 fluorescent labels, the gene is targeted by several dsDNA polynucleotide probes. Both probes are applied together, in the same hybridisation mixture.

Probe design:

To target viruses hunting Altiarchaeota, probes were design by Cristina Moraru (gene-prober.icbm.de/).

Perform virusFISH with fresh flocks

Flocks= Samples fixed immediate at the sampling site using a final concentration of 3% methanol-free formaldehyde

IBIDI glass slide 35 mm Grid 500 glass Bottom

<u>Preparation prior to the experiment (details see virusFISH protocol):</u>

-hybridisation (HYB) buffer (store at -20 degrees celsius)

-virus targeting probe mix stock (store at -20 degrees celsius)

-16S rRNA probe stock (store at -20 degrees celsius)

-DAPI solution (store at 4 or -20 degrees celsius)

Preparation of hybridisation mix (HYB mix):

90 uL HYB mix prepared by mixing 88 uL HYB buffer (prepared beforehand as described in the virusFISH protocol) with 0.9 uL SM probe and 1.08 uL virus probe (prepared previously) (the virus probe mix volume depends on the results of the stock preparation)

Preparation hybridisation chamber (in fumehood):

For 20% Formamide (FA) (depends on probes): in HYB chamber mix 4 mL water 1 mL Formamide first mix in chamber then add paper tissue

Preparation of washing buffer (WB): (depends on FA concentration, 3x the volume required to merge dish(es))

2.15 mL 5 M NaCl

1 mL Tris 1M

0.5 mL EDTA 0.5 M

fill up with water to 50 mL

25 uL 20% SDS

The WB step and the PBS step will be performed in a glassbox designed to place glass slides inside with a upright position. Glas slides are used to place the ibidi dished upright in the Buffer, to increase the liquid exchange in the well

Caco= cacodylate buffer

GA= glutardialdehyde for EM

After FM imaging:

Cacofixation with GA 2.5%

2.5%*2 mL= x mL * 50% = 100 ul GA in 1.9 mL Caco

Please make yourself familiar with the SDS of the used chemicals, as some of them are toxic or

carcinogenic

Steps

- Switch ovens on (85 and 46 degrees)
- prepare HYB mix (fume hood)
- deposit flocks on the grid
- prepare hyb chamber (fumehood)
- add HYB mix onto slide once the EtOH barely evaporated (fumehood)
- place dishes in hyb chamber close lid (fume hood)
- 30 min 85 degrees (oven)
- transfer to second oven 3 h 46 degrees
- prepare WB
- preheat WB in water bath (48 degrees)
- prewash wells with a small portion WB (fume hood)
- merge dishes in WB 15 min 48 degrees (water bath)
- wash 20 min in 1XPBS
- dip 1 min in ice cold milliq water
- dip 2 times in EtOH
- cover in Dapi solution 4 µg/mL for 3 min
- wash in milliq water
- fill dish with 1-2 mL caco, (same concentration/buffer used during sample preparation after fixation)
- close dish with lid and parafilm
- Perform fluorescence microscopy (easiest if inverted microscope)
- Exchange Caco buffer for Post fixation in Caco 2.5% GA 30 min
- Store in fresh Caco in fridge over night

Unique eLabID: 20230222-b657f4ca0d5b01542e8f2cc51f048b80e8d227b1
Link: https://elabftw.cvis.uni-due.de/experiments.php?mode=view&id=4895
