# Supplementary material for: Spatio-functional organization in virocells of small uncultivated archaea from the deep biosphere
Source: ISME J. 2023 Jul 19;17(10):1789–92. doi: 10.1038/s41396-023-01474-1 (PMC10504349; doi:10.1038/s41396-023-01474-1)
Supplement: Supplementary file 3 — Supplementary Information 3 [file 41396_2023_1474_MOESM3_ESM.pdf]

SEM preparation on slide with Acetone

Date: 2023-02-22
Tags: EM
Created by: Indra Banas

Established for MSI Biofilms.

This workflow is the second part of the CLEM sample preparation.

It starts after:

- all FM images have been taken, while the dish filled with buffer (75 mM cacodylate buffer (2 mM $MgCl_2$, pH 6.5-7))

- the buffer has been exchanged for GA fixation (2.5% in cacodylate buffer 30 min)

Some considerations:

Work in a fumehood until transfer to critical point drying

Caco= cacodylate buffer

-After attachment of the biofilm to the slide, drying of the biofilm should be absolutely avoided.

-The single steps mean: Remove the old buffer carefully and add the listed solution to cover the slide, incubate for the respective time

-The molarity of the buffer depends on the sample

-Incubation times are a recommendation, the incubation time for osmium tetroxide is critical

-1% osmium tetroxide in Caco is prepared freshly just before use from a 4% osmium tetroxide in water stock. (keep on Ice the whole time)

-Osmium is recommended with an acetone dehydration step, an alternative might be replacing acetone with ethanol

-until the glass coverslip is detached from the dish, the dish can be covered wit the delivered lid

-the detachment must be performed before adding acetone, as it dissolves the dish.

-with some training a diamond pen can be used to cut out a circle of the glass coverslip

-transfer the coverslip into a glass petri dish or similar

-the listed amount of acetone is mixed with deionised water, use an excess of liquid and cover during incubation, to minimize evaporation

-Better use an excess of acetone water mixture and cover

Please make yourself familiar with the SDS of the used chemicals, as some of them are toxic or carcinogenic

Steps

- 5 min Caco
- 20 min Caco
- 30 min Caco
- 40 min Caco
- 30 min 1% Osmium in Caco
- 5 min Caco
- 15 min Caco
- 5 min water
- 15 min water
- detach the glass coverslip from the dish
- 25 min water
- 10 min 10% Acetone
- 10 min 20% Acetone
- 15 min 40% Acetone
- 10 min 60% Acetone
- 15 min 80% Acetone
- 5 min 100% Acetone
- 15 min 100% Acetone (anhydrous)
- 100% Acetone (anhydrous) over night
- Critical point drying
- Sputter coating with 2 nm Pt/Pd

Unique eLabID: 20230222-5b33a6b51d0f24f66adccc0fadc790ad718d9254
Link: https://elabftw.cvis.uni-due.de/experiments.php?mode=view&id=4898
